# Diagnosis of Alzheimer’s Disease Using Brain Network

**DOI:** 10.3389/fnins.2021.605115

**Published:** 2021-02-05

**Authors:** Ramesh Kumar Lama, Goo-Rak Kwon

**Affiliations:** The Alzheimer’s Disease Neuroimaging Initiative, Department of Information and Communication Engineering, Chosun University, Gwangju, South Korea

**Keywords:** Alzhieimer’s disease, brain network, node2vec, extreme learning machine, support vector machine

## Abstract

Recent studies suggest the brain functional connectivity impairment is the early event occurred in case of Alzheimer’s disease (AD) as well as mild cognitive impairment (MCI). We model the brain as a graph based network to study these impairment. In this paper, we present a new diagnosis approach using graph theory based features from functional magnetic resonance (fMR) images to discriminate AD, MCI, and healthy control (HC) subjects using different classification techniques. These techniques include linear support vector machine (LSVM), and regularized extreme learning machine (RELM). We used pairwise Pearson’s correlation-based functional connectivity to construct the brain network. We compare the classification performance of brain network using Alzheimer’s disease neuroimaging initiative (ADNI) datasets. Node2vec graph embedding approach is employed to convert graph features to feature vectors. Experimental results show that the SVM with LASSO feature selection method generates better classification accuracy compared to other classification technique.

## Introduction

Alzheimer’s disease (AD), which causes majority of dementia is a progressive neurodegenerative disease (American Psychiatric Association, 1994; Liu F. et al., 2014; Schmitter et al., 2015; Alzheimer’s association, 2016). The subtle AD neuropathological process begins years before the visible progressive cognitive impairment, which is trouble to remember and learn new information. Currently there is no cure and treatment to slow or stop its progression. Currently, more research works are focused toward earlier intervention of AD. Thus accurate diagnosis of disease at its early stage makes great significance in such scenario.

With the availability of recent neuroimaging technology, promising result is obtained in the early and accurate detection of AD (Hanyu et al., 2010; Górriz et al., 2011; Gray et al., 2012). The study of progression of disease and early detection is carried out by using different imaging models, such as electroencephalography (EEG) (Pfefferbaum et al., 2000), functional magnetic resonance imaging (fMRI) (Masliah et al., 1993), single-photon emission computed tomography (SPECT) (Chen et al., 2013) and positron emission tomography (PET) (Ly et al., 2014).

Similarly, structural magnetic resonance imaging (MRI) (Hanyu et al., 1999; Canu et al., 2010; Bendlin et al., 2012) is the most commonly used imaging system for study of AD. The feature extracted from MRI is typically gray matter volumes and measured as important biomarker for the study of neurodegeneration, alterations of hippocampal white matter pathways is often observed in AD (Delbeuck et al., 2003; Liu Y. et al., 2014). Several studies reveal the alterations in widely distributed functional and structural connectivity pairs are prevalent in AD and mild cognitive impairment (MCI) (Delbeuck et al., 2007; Acosta-Cabronero et al., 2012). Additionally, in recent studies, the resting-state functional magnetic resonance imaging (rs-fMRI) has been widely used for the investigations of progression of AD (Stam et al., 2006; Sorg et al., 2007; Supekar et al., 2008; Doan et al., 2017). This imaging system evaluates the impulsive variabilities seen in the blood oxygenation level-dependent (BOLD) indications in various regions of the brain. Several studies are carried out based on aberrant regional spontaneous fluctuation of BOLD, functional connectivity and alteration in functional brain network. These studies are carried out in different networks, such as default mode network, somatomotor network, dorsal attention network, limbic network, and frontoparietal control network (Bullmore and Sporns, 2009). Thus, the graph theory based network analyses of human brain functional connectomes, provides better insights of the network structure to reveal abnormal patterns of organization of functional connectivity in AD infected brain (Rubinov and Sporns, 2010; Sporns, 2011).

Graph theory is a mathematical approach to study complex networks. Network is constructed of vertices which are interconnected by edges. Vertices in our case are brain regions. Graph theory is widely used as tool for identifying anatomically localized subnetworks associated with neuronal alterations in different neurodegenerative diseases (Bajo et al., 2010). In fMRI images, graph represents causal relations or correlations of different nodes in constructed networks. However, the brain network built by graph has non-Euclidian characteristics. Thus, applying machine learning techniques to analyze the brain networks is challenging. We use graph embedding to transform graphs to a vector or set of vectors to overcome this problem. Embedding captures the graph topology, vertex-vertex relationship, and other relevant graph information. In the current study, we used node2vec graph embedding technique to transform vertex and edge of brain network graph to feature vector. With the help of this model we have analyzed and classified the networks of brain from fMRI data into AD, MCI, and HC.

## Materials for the Study

### fMRI Dataset

In our study, we have used the dataset from Alzheimer’s disease neuroimaging initiative database (ADNI)^1^. The ADNI database was launched in 2004. The database consists of subjects of age ranging from 55–90 years. The goal of ADNI is to study the progression of the disease using different biomarkers. This includes clinical measures and assesses of the structures and functions of brain for the course of different disease states.

All participants were scanned using 3.0-Telsa Philips Achieva scanners at different centers. Same scanning protocol were followed for all participants and the set parameters were ratio of Repetition Time (TR) to Echo Time (TE) i.e., TR/TE = 3000/30 *ms*, 140 volumes, also voxel thickness as 3.3 mm, acquisition matrix size = 64 × 64, 48 slices, flip angle = 80° Similarly, 3D T1-weighted images were collected using MPRAGE sense2 sequences with acquisition type 3D, field strength = 3 Tesla, flip angle 9.0 degree, pixel spacing X = 1.0547000169754028 mm; Pixel Spacing Y = 1.0547000169754028 mm, slice thickness = 1.2000000476837158 mm; echo time (TE) 2.859 ms, inversion time (TI) 0.0 ms, repetition time (TR) 6.6764 ms and weighting T1. We selected subjects as specified in Table.

### Subjects

We selected 93 subjects from ADNI2 cohort. The purpose of ADNI2 is to examine how brain imaging and other biomarkers can be used to measure the progression of MCI and early AD. The ADNI selects and categorizes participants in specific group based on certain inclusion criteria. The criteria are well defined in^2^. We selected the subjects according to availability of both MRI and fMRI data. Thus, the subjects with following demographic status as shown in Table 1 with following average age, clinical dementia rating (CDR) and mini-mental state estimation (MMSE) out of all available data in ADNI2 cohort were selected in our study.

**TABLE 1:** Summary of subject’s demographic status.

**Number of**	**HC (*n* = 31)**	**MCI (*n* = 31)**	**AD (*n* = 31)**
	
**subjects**	**Mean (SD)**	**Mean (SD)**	**Mean (SD)**
Age (years)	73.9 ± 5.4	74.5 ± 5.0	SD = 72.7 ± 7.0
Global CDR	0.04 ± 0.13	0.5 ± 0.18	0.95 ± 0.30
MMSE	28.9 ± 1.65	27.5 ± 2.02	20.87 ± 3.6

1. 31 HC subjects: 14 males, 17 females; age ± SD = 73.9 ± 5.4 years with the mini-mental state estimation (MMSE) score of 28.9 ± 1.65 and the range was 24–30.
2. 31 MCI subjects: 17 males, 14 females; age ± SD = 74.5 ± 5.0 with the MMSE score of 27.5 ± 2.02, and range was 22–30.
3. 31 AD subjects: 13 males, 18 females; age ± SD = 72.7 ± 7.0 with MMSE = 20.87 ± 3.6, and the range was 14–26.

### Data Preprocessing

We used data processing subordinate for the resting state fMRI via DPARSF^3^ (Chao-Gan and Yu-Feng, 2010) and the statistical parametric mapping platform via SPM8^4^ aimed at the preprocessing of rs-fMRI data. All the images initially obtained from scanner were in the format of digital imaging and communications in medicine (DICOM). We converted these images to neuroimaging informatics technology initiative (NIfTI) file format. Signal standardization and participant’s adaptation to the noise while scanning each participant are carried out by discarding the first 10 time points for each participant. Next, we preformed preprocessing operation through following steps:

For slice-timing correction last slice was referred reference slice. Friston 24-parameter model with 6 parameters of head motion, 6 parameters of head motion from the previous time point, and 12 corresponding squared items were employed for realignment for head movement compensation. Similarly, after the realignment, individual structural images (T1-weighted MPRAGE) were registered to the mean functional image. For the standardization of the rs-fMRI toward the original place was accomplished with the help of diffeomorphic anatomical registration through exponentiated lie algebra (DARTEL) as in Ashburner (2007) (resampling voxel size = 3 mm × 3 mm × 3 mm). A 6 mm full width at half-maximum (FWHM) Gaussian kernel spatial smoothing was employed for the smoothing. Next, we performed linear trend exclusion and also the temporal band pass filtering which ranges at (0.01 Hz < f < 0.08 Hz) on the time series of each voxel. Finally, cerebrospinal as well as white matter signals along with six head-motion parameters were regressed out to reduce the effects of nuisance signals.

### Proposed Framework

**FIGURE 1:**
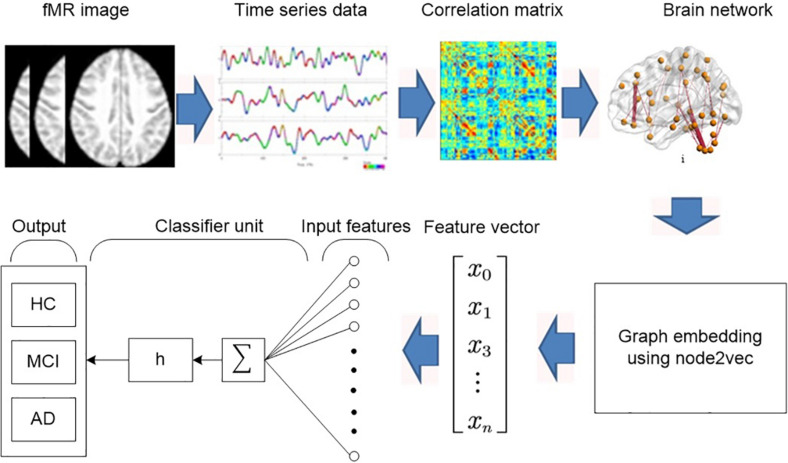
Block diagram of the proposed diagnosis system.

This proposed method consists of the following four major functional steps as shown in Figure 1:

1. Construct a brain network using graph theory.
2. Convert graph to feature vector using node2vec graph embedding.
3. Reduce the features.
4. Perform the classification using regularized extreme learning machine (RELM) and linear support vector machine (LSVM).

### Construction of Brain Networks

For the construction of network from fMR images, we first preprocessed the raw fMR data as described in data preprocessing section. Next, we used the automated anatomical labeling (AAL) atlas to identify the brain regions of interest (ROI). The whole image was divided in 116 regions with each hemisphere. Next, we calculate the average time series of each ROI for each subject by averaging their time series across the voxels within each ROI. For each subject, a matrix of 130 rows and 116 columns was obtained. In the matrix, every row denotes the time series conforming to a give ROI, while information of total regions at a definite time point are stored at each column. The mean time series of each brain region were obtained for each individual by averaging the time series within the region. For *L*_*i*_ = (*l*_*i*_(1),*l*_*i*_(2),…,*l*_*i*_(*n*)) and *L*_*j*_ = (*l*_*j*_(1),*l*_*j*_(2),…,*l*_*j*_(*n*)) are two *n* length time series of brain region *i* and *j*, the Pearson’s correlation (PC) between them can be calculated as

(1) $$P\,C_{i\,j}=\frac{c\,o\,v\,\left(L_{i},L_{j}\right)}{\sigma_{L_{i}}\,\sigma_{L_{j}}}$$

Where *cov*(*L*_*i*_,*L*_*j*_) is covariance of variables *L*_*i*_ and *L*_*j*_. Similarly, σ_*L_i*_ and σ_*L_j*_ are standard deviation of variables *L*_*i*_ and *L*_*j*_. This operation results into 116 × 116 correlation matrix which defines the relation amongst different regions of brain and matches to the functional connectivity network.

### Graph-Embedding

Graphs are complex data structures, consisting a finite set of vertices and set of edges which connect a pair of nodes. One of the possible solutions to manipulate prevalent pattern recognition algorithms on graphs is embedding the graph into vector space. Indeed, graph embedding is a bridge between statistical pattern recognition and graph mining. We employ the node2vec (Grover and Leskovec, 2016) algorithm as graph embedding tool in this study. The node2vec algorithm aims to learn a vectorial representation of nodes in a graph by optimizing a neighborhood preserving objective. It extends the previous node embedding algorithm Deepwalk (Canu et al., 2010) and it is inspired from the state of art word embedding algorithm word2vec (Delbeuck et al., 2003).

In word2vec, given a set of sentences also known as corpus, the model learns word embedding by analyzing the context of each word in the body. The word2vec uses the neural network with one hidden layer to transform words into embedding vectors. This neural network is known as Skip-gram. This network is trained to predict the neighboring word in the sentence. It accepts the word at the input and is optimized such that it predicts the neighboring words in a sentence with high probability.

node2vec applies the same embedding approach to train and predict the neighborhood of a node in graph. However, word is replaced by the node and the bag of nodes is used instead of corpus. The sampling is used to generate this bag of nodes from a graph. Generally, the graph embedding consists of three steps:

#### Sampling

**FIGURE 2:**
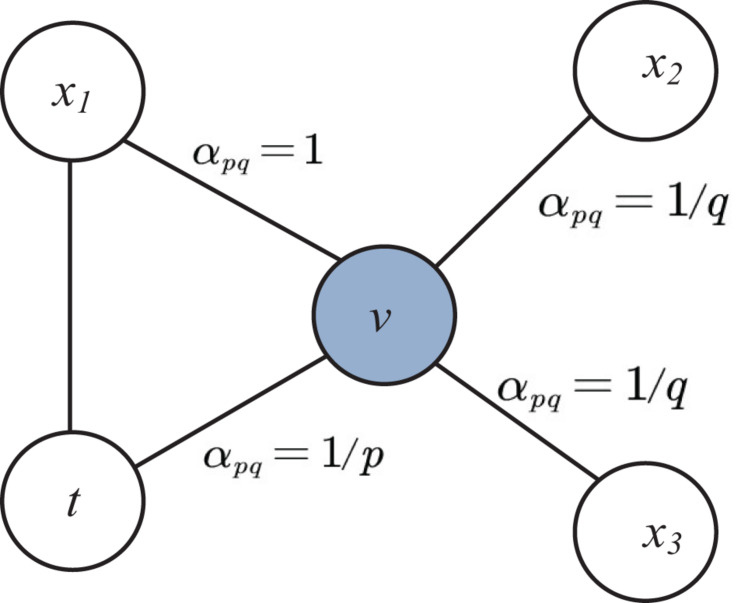
Illustration of node selection in node2vec algorithm.

A graph is sampled with random walks. This random walk results in bag of nodes of neighborhood from sampling. The bag of nodes acts as a collection of contexts for each node in the network. The innovation of node2vec with respect to Deepwalk is the use of flexible biased random walks on the network. In Deepwalk, random walk is obtained by a uniform random sampling over the linked nodes, while node2vec combine two different strategies for the network exploration: depth-first search (DFS) and breadth-first-search (BFS). For current random walk position at node *v* and traversed position at previous step at node *t* and neighboring nodes *x*_*1*_, *x*_*2*_ and *x*_*3*_, the sampling of next node *x* is determined by evaluating the unnormalized transition probabilities π_*vx*_ on edge (*t*,*v*) with the static edge weight *w*_*vx*_ as shown in Figure 2. This unnormalized transition probability is estimated based on search bias α defined by two parameters *p* and *q* such that π_*vx*_ = α_*pq*_(*t*,*x*)⋅*w*_*vx*_ where.

(2) $$\alpha_{p\,q}\,\left(t,x\right)=\left\{ \begin{array}{ccc} \frac{1}{p}, & \text{if} & d_{t\,x}=0\\ 1, & \text{if} & d_{t\,x}=1\\ \frac{1}{q}, & \text{if} & d_{t\,x}=2 \end{array}\right.$$

Here *d*_*tx*_ denotes the shortest path distance between nodes *t* and *x*.

The parameter *p* determines the likelihood of sampling the node *t* again during random walk. When the value of *p* is high revisit of the node possibility is low. Similarly the parameter *q* allows to different between local and global nodes. If *q* > 1, the random walk has the likelihood of sampling the nodes around the node *v* is high.

#### Training Skip-Gram

The bag of nodes generated from the random walk is fed into the skip-gram network. Each node is represented by a one-hot vector and maximizes the probability for predicting neighbor nodes. The one-hot vector has size same as the size of the set of unique words used in the text corpus. For each node only one dimension is equal to one and remaining are zeros. The position of dimension having one in vector defines the individual node.

#### Computing Embedding

The output of the hidden layer of the network is taken as the embedding of the graph.

### Feature Reduction Techniques

#### Support Vector Machine-Recursive Feature Elimination (SVM-RFE)

Support vector machine-recursive feature elimination is a multivariate feature reduction algorithm is based on wrapper model. This method is recursive and in each of iteration of the RFE, LSVM model is trained. This method starts by constructing a model on the complete set of features and computing the importance score for each feature. The least important features are removed and the model is rebuilt and the importance scores are again computed. This recursive procedure is continued until all the features are eliminated. Then, the features are ranked according to the order of elimination. A detailed description of SVM-RFE procedure presented in a previous paper (Guyon et al., 2002). In this work, after applying SVM-RFE, the most significant training features that make the most of cross-validated accurateness are kept to train the classifiers.

#### Least Absolute Shrinkage and Selection Operator (LASSO)

Least absolute shrinkage and selection operator (Tibshirani, 1996) is a powerful method which is used to remove insignificant features. Two major tasks of this method are regularization and feature selection. This method minimizes residual sum of squares of the model using ordinary least square regression (OLS) by placing a constraint on the sum of the absolute values of the model parameters. LASSO computes model coefficients β by minimizing the following function:

$$R\,S\,S_{L\,A\,S\,S\,O}\,\left(\beta_{i},\beta_{0}\right)$$

(3) $$\;=\begin{array}{cc} \arg\min & \\ \beta & \end{array}\,\left[\sum_{i=1}^{n}{\left(y_{i}-\left(\beta_{i}\,x_{i}+\beta_{0}\right)\right)}^{2}+\alpha \,\sum_{j=1}^{k}\left|\beta_{j}\right|\right]$$

Where, *x*_*i*_ is the graph embedded feature input data, a vector of *k* values at observation *j*, and *n* is the number of observations. *y*_*i*_ is the response at observation *i*. α is a non-negative user defined regularization parameter. This parameter controls the strength of penalty. When α is sufficiently large then coefficients are forced to be zero which leads to produce few relevant features. If α approaches 0 the model becomes OLS with more relevant features (Hanyu et al., 2010).

#### Features Selection With Adaptive Structure Learning (FSASL)

Features selection with adaptive structure learning is an unsupervised method which performs data manifold learning and feature selection. This method first utilizes the adaptive structure of the data to construct the global learning and the local learning. Next, the significant features are selected by integrating both of them with *L*_*2,1*_-norm regularizer. This method utilizes the sparse reconstruction coefficients to extract the global structure of data for global learning. In sparse representation, each data sample *x*_*i*_ can be approximated as a linear combination of all the other samples, and the optimal sparse combination weight matrix.

For local learning, this method directly learns a Euclidean distance induced probabilistic neighborhood matrix (Du and Shen, 2015).

$$\min_{W,S,P}\left({∥W^{T}\,X-W^{T}\,X\,S∥}^{2}+\alpha \,{∥S∥}_{1}\right)$$

$$\;+\beta \,\sum_{i,j}^{n}\left({∥W^{T}\,x_{i}-W^{T}\,x_{j}∥}^{2}\,P_{i\,j}+\mu \,P_{i\,j}^{2}\right)+\gamma \,{∥W∥}_{21}$$

(4) $$s.t.S_{i\,i}=0,P\,1_{n}=1_{n},P\geq 0,W^{T}\,X\,X^{T}\,X=I$$

Where, α is used to balancing the sparsity and the reconstruction error, β and γ are regularization parameters for global and local structure learning in first and second group and the sparsity of feature selection matrix in the third group. Similarly, *S* is used to guide the search of relevant global feature and *P* defines the local neighborhood of data sample *x*_*i*_.

#### Local Learning and Clustering Based Feature Selection (LLCFS)

LLCFS is clustering based feature selection method. This method learns the adaptive data structure with selected features by constructing the k-nearest neighbor graph in the weighted feature space (Zeng and Cheung, 2011). The joint clustering and feature weight learning is performed by solving the following problem.

$$\min_{Y,{\left\{W^{i},b^{i}\right\}}_{i=1}^{n},z}\sum_{i=1}^{n}\sum_{c^{\prime }=1}^{c}[\sum_{x_{j}\in N_{x_{i}}}\beta {\left(Y_{i\,c^{\prime }}-x_{j}^{T}W_{c^{\prime }}^{i}-b_{c}^{i}\right)}^{2}$$

$$+{\left(W_{c^{\prime }}^{i}\right)}^{T}\mathrm{diag}\left(z^{-1}\right)W_{c^{\prime }}^{i}]$$

(5) $$s.t.1_{d}^{T}\,z=1,z\geq 0$$

Where *z* the feature weight vector and *N*_*x_i*_ is the k-nearest neighbor of *x*_*i*_ based on *z* weighted features.

#### Pairwise Correlation Based Feature Selection (CFS)

CFS selects features based on the ranks attributes according to an empirical evaluation function based on correlations (Hall, 2000). Subsets made of attribute vectors are evaluated by evaluation function, which are associated with the labels of class, however autonomous among each another. CFS accepts that unrelated structures express a low correspondence with the class and hence they are ought to be overlooked by the procedure. Alternatively, additional features must be studied, as they are typically hugely correlated with one or additional amount of other features.

#### Classification

Two of the prevalent machine-learning classification algorithms namely, LSVM, and RELM are studied in this article. The results acquired through the experiments of these classifiers show that RELM classifier performs better than others respective of the computation time required and accuracy value. Each of the methods is described in brief in the subsections below.

#### Support Vector Machine Classifier

Linear support vector machine (Cortes and Vapnik, 1995) is principally a supervised binary classifier that classifies separable and non-separable data. This type of classification is usually used in the field of neuroimaging and is deliberated as one of the finest machine-learning method in the domain of the neuroscience for past decades. It discovers the best hyperplane to split both classes which has optimum boundary from support vectors for the duration of the training. The classifier decides on the basis of the estimated hyperplane to test the new data points. For the patterns that are linearly separable, LSVM can be used. Alternatively, LSVM is not capable of guaranteeing improved performance in the complex circumstances with the patterns that are not separable. In such circumstances, Kernel trick is used to extend the LSVM. The input arrays of linear SVM are plotted to the space dimensions using the kernels. Both the linear as well as non-linear radial basis function (RBF) kernels are extensively trained using SVM kernels.

#### Extreme Learning Machine

ELM (Extreme Learning Machine) is single layer feedforward neural networks (Huang et al., 2006; Zhang et al., 2015). This neural network is implemented using Moore-Penrose generalized inverse to set its weights (Peng et al., 2013; Cao et al., 2016). Thus, this learning algorithm doesn’t require iterative gradient-based backpropagation to tune the artificial hidden nodes. Thus this method is considered as effective solution with extremely reduced complexity (Cambria and Huang, 2013; Qureshi et al., 2016). ELM with *L* number of hidden nodes and g(x) as activation function is expressed as

(6) $$Y_{L}\,\left(x\right)=\sum_{i=1}^{L}\beta_{i}\,h_{i}\,\left(x\right)=h\,\left(x\right)\,\beta_{i}$$

Where *x* is an input vector. *h*_*i*_(*x*) is the input to output node from hidden node output. β = [β_1_,……,β_2_]*T* is the weight matrix of *i*^th^ node. The input weight *w*_*i*_ and the hidden layer biases *b*_*i*_ are generated randomly before the training samples are acquired. Thus iterative back-propagation to tune these parameters is not needed. Given N training samples ${\left\{\left(x_{j},t_{j}\right)\right\}}_{j=1}^{N}$. The objective function of ELM is expressed as,

(7) $$∥H\left(w_{1},\ldots .w_{\tilde{N}},b_{1},\ldots .,b_{\tilde{N}}\right)\hat{\beta }-T∥=\min_{\beta }∥H\hat{\beta }-T∥$$

with

$$H\left(w_{1},\ldots .w_{\tilde{N}},b_{1},\ldots .,b_{\tilde{N}}\right)$$

(8) $$=\left[\begin{array}{ccc} g\left(w_{1}.x_{1}+b_{1}\right)\cdots g\left(w_{L}.x_{1}+b_{L}\right) & & \\ \vdots \,\cdots \,\vdots & & \\ g\left(w_{1}.x_{N}+b_{1}\right)\cdots g\left(w_{L}.x_{N}+b_{L}\right) & & \end{array}\right]$$

$$,\beta =\left[\begin{array}{c} \beta_{1}^{T}\\ \vdots \\ \beta_{L}^{T} \end{array}\right]T=\left[\begin{array}{c} t_{1}^{T}\\ \vdots \\ t_{L}^{T} \end{array}\right]$$

Here *H* represents the hidden layer output matrix and *T* represents output label of training data matrix. The output weight matrix β is calculated as

(9) $$\beta =H^{+}\,T$$

Here, *H*^+^ represents the Moore-Penrose generalized inverse of the matrix *H*. Since ELM learning approach requires no backpropagation, this method is best suited for the binary and multiclass classification of big data and neuroimaging features. However the decrease in computation time comes with the expense of increase in the error in the output, which ultimately decreases the accuracy. Thus, a regularization term is added to improve generalization performance and make the solution more robust. The output weight of the regularized ELM can be expressed as

(10) $$\beta ={\left(\frac{I}{C}+H^{T}\,H\right)}^{-1}\,H^{T}\,T$$

#### Performance Evaluation

We evaluated the performance using the SVM and RELM classifiers for each specific test including the binary and multiclass test. Confusion matrix is constructed to visualize the performance of the binary classifier in a form of a as shown in Table 2. Correct numbers of prediction of classifier are placed on the diagonal of the matrix. These components are further divided into true positive (TP), true negative (TN), which represent correctly identified controls. Similarly, the false positive (FP) and false negative (FN) represent the number of wrongly classified subjects.

**TABLE 2:** Confusion matrix.

**Accurate Class**	**Predicted Class**	
	**C1**	**C2**
C1	TP	FN
C2	FP	TN

The proportion of subjects which are correctly classified by the classifier is expressed as the accuracy.

(11) $$A\,C\,C=\frac{T\,P+T\,N}{T\,P+T\,N+F\,P+F\,N}$$

However, for dataset with unbalanced class distribution accuracy may be a good performance metric. Thus two more performance are used. These metrics are known as sensitivity and specificity are used.

(12) $$S\,E\,N=\frac{T\,P}{T\,P+F\,N}$$

(13) $$S\,P\,E=\frac{T\,N}{T\,N+F\,P}$$

The sensitivity (SEN) measures the rate of true positives (TP) while the specificity (SPE) measures rate of true negatives (TN).

## Results

### Demographic and Clinical Findings

We did not find a significant group difference in age in AD versus HC, AD versus MCI and MCI versus HC. However significant group difference was found in MMSE (*P* < 0.01) and CDR (*P*<0.01) in all group combinations. The gender proportion on both AD and HC is male dominant. AD has 54.83% and HC has 45.16% male dominance. Table 1 shows the detailed descriptions and analysis of these variables.

### Classification Results

**FIGURE 3:**
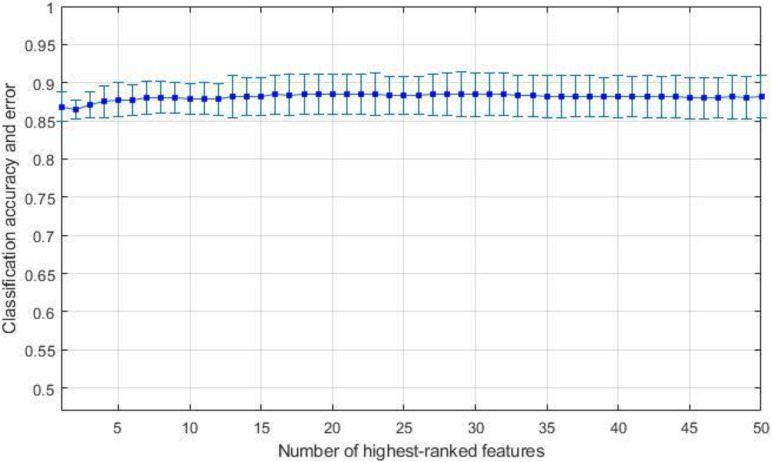
Average accuracy and standard deviation for AD against HC using RELM classification method on reduced datasets using LASSO feature selection.

We have observed the performance of our proposed algorithm and compared it with that of the RELM classifier and LSVM classifier for respective test comprising the binary classification. The performance shown by the binary classifier is envisaged as a confusion matrix as presented in Table 1. Elements on the diagonal elements of the matrix specify the accurate estimations by the classifier. These elements are further divided as true positive (TP) and true negative (TN), which signifies appropriately recognized controls. Correspondingly, all the erroneously classified matters can be symbolized by false positive (FP) and false negative (FN). We evaluated the feature selection and classification algorithms on data set using a 10-flold cross validation (CV). First, we divided the subjects into 10 equally sized subsets: each of these subsets (folds), containing 10% of the subjects as test set and remaining 90% for training set. Then feature ranking was performed on the training sets. We used different algorithms to rank the features. Linear SVM and RELM classifiers were trained using these top-ranked features. For each training and test we performed separate feature selection to avoid the feature selection bias during 10-fold cross validation. We calculated cross validated average classification accuracy and standard deviation for specific feature using *k*-top most ranked features, where *k* ranges from 1 to 50. We repeatedly tested for 5 iterations and plotted the mean accuracy and standard deviation as shown in Figure 3 for LASSO feature selection and RELM classifier. Finally, we calculated the mean accuracy and standard deviation of highest ranked features for different feature selection and classification methods as depicted in Tables 3–8 and the bold values in each table indicate the maximum value of accuracy, sensitivity and specificity. Maximum and minimum value of accuracy, sensitivity and specificity are calculated amongst corresponding values estimated for highest ranked features as shown in Figure 3.

**TABLE 3:** 10-fold cross-validation binary mean classification performance for AD against HC using RELM classifier using different feature selection methods.

**Feature selection method**	**Performance metrics**	**Mean (%)**	**Standard deviation**	**Max (%)**	**Min (%)**
LASSO	Accuracy	**87.723**	0.468	88.663	85.82
	Sensitivity	**90.93**	0.341	91.525	89.50
	Specificity	**84.52**	0.792	85.891	82.14
	F-measure	0.883			
FSASL	Accuracy	76.181	1.069	78.551	73.45
	Sensitivity	76.233	1.255	78.839	72.58
	Specificity	75.664	1.264	77.868	72.20
	F-measure	0.785			
LLCFS	Accuracy	75.737	1.004	78.690	71.64
	Sensitivity	74.205	1.069	77.031	70.11
	Specificity	77.881	1.378	81.036	73.64
	F-measure	0.817			
CFS	Accuracy	80.517	1.737	82.86	74.005
	Sensitivity	80.035	1.813	82.22	73.25
	Specificity	79.16	1.977	81.79	73.084
	F-measure	0.867			
SVM-RFE	Accuracy	68.57	1.186	70.474	65.60
	Sensitivity	75.99	1.676	78.832	71.715
	Specificity	60.92	1.301	63.426	57.34
	F-measure	0.6743			

**TABLE 4:** 10-fold cross-validation binary mean classification performance for HC against MCI using RELM classifier using different feature selection methods.

**Feature selection method**	**Performance metrics**	**Mean (%)**	**Standard deviation**	**Max (%)**	**Min (%)**
LASSO	Accuracy	**96.11**	0.859	96.88	91.33
	Sensitivity	**95.03**	1.080	95.93	89.84
	Specificity	**97.18**	0.798	97.84	92.93
	F-measure	0.973			
FSASL	Accuracy	85.85	0.9129	87.503	80.88
	Sensitivity	79.27	0.986	81.484	76.01
	Specificity	92.03	1.4433	93.308	85.40
	F-measure	0.937			
LLCFS	Accuracy	82.29	0.624	83.39	80.77
	Sensitivity	77.54	0.73	78.566	75.408
	Specificity	86.81	1.081	88.41	83.74
	F-measure	0.85			
CFS	Accuracy	80.67	1.68	90.427	74.48
	Sensitivity	88.43	2.328	74.968	80.49
	Specificity	72.38	1.3677	74.486	68.58
	F-measure	0.795			
SVM-RFE	Accuracy	80.20	0.920	82.001	77.89
	Sensitivity	84.00	1.207	85.99	79.86
	Specificity	75.91	1.251	77.806	73.29
	F-measure	0.815			

**TABLE 5:** 10-fold cross-validation binary mean classification performance for MCI against AD using RELM classifier using different feature selection methods.

**Feature selection method**	**Performance metrics**	**Mean (%)**	**Standard deviation**	**Max (%)**	**Min (%)**
LASSO	Accuracy	**93.86**	0.766	94.90	89.128
	Sensitivity	**91.93**	0.757	93.67	89.836
	Specificity	**95.92**	1.204	96.61	88.580
	F-measure	0.968			
FSASL	Accuracy	85.358	1.030	86.76	80.29
	Sensitivity	85.088	0.951	86.61	80.47
	Specificity	85.201	1.4227	86.829	79.27
	F-measure	0.879			
LLCFS	Accuracy	90.32	1.06316	91.88	86.50
	Sensitivity	93.33	2.0782	95.63	87.43
	Specificity	87.49	0.9471	89.56	85.75
	F-measure	0.895			
CFS	Accuracy	79.13	1.2768	81.595	75.10
	Sensitivity	83.59	1.2281	85.60	78.94
	Specificity	74.83	1.7084	78.927	70.54
	F-measure	0.795			
SVMRFE	Accuracy	77.5974	0.8177	78.93	74.98
	Sensitivity	76.802	1.1299	79.225	73.26
	Specificity	78.182	0.8359	79.289	75.44
	F-measure	0.8169			

**TABLE 6:** 10-fold cross-validation binary mean classification performance for AD against HC using LSVM classifier using different feature selection methods.

**Feature selection method**	**Performance metrics**	**Mean (%)**	**Standard deviation**	**Max (%)**	**Min (%)**
LASSO	Accuracy	**90.63**	0.515	91.51	88.52
	Sensitivity	87.044	0.585	88.03	85.44
	Specificity	**94.315**	0.671	95.35	90.95
	F-measure	0.958			
FSASL	Accuracy	82.895	1.4020	85.60	80.19
	Sensitivity	78.206	1.5118	81.99	75.21
	Specificity	87.712	1.7666	90.02	82.85
	F-measure	0.8360			
LLCFS	Accuracy	81.19	1.438	83.37	77.68
	Sensitivity	85.15	2.087	88.068	80.49
	Specificity	76.39	1.25	78.983	73.65
	F-measure	0.8095			
CFS	Accuracy	88.37	1.78	91.18	83.25
	Sensitivity	**87.95**	1.72	91.98	84.52
	Specificity	88.79	2.17	90.71	81.28
	F-measure	0.903			
SVMRFE	Accuracy	65.99	1.48	68.51	62.05
	Sensitivity	65.03	1.41	67.73	61.46
	Specificity	67.40	2.327	70.53	61.27
	F-measure	0.671			

**TABLE 7:** 10-fold cross-validation binary mean classification performance for HC against MCI using LSVM classifier using different feature selection methods.

**Feature selection method**	**Performance metrics**	**Mean (%)**	**Standard deviation**	**Max (%)**	**Min (%)**
LASSO	Accuracy	**98.91**	0.456	99.25	95.82
	Sensitivity	**99.68**	0.56	100.0	95.48
	Specificity	**98.11**	0.46	98.51	96.00
	F-measure	0.9856			
FSASL	Accuracy	81.28	1.010	83.01	77.64
	Sensitivity	84.61	1.389	86.62	79.81
	Specificity	77.92	1.121	79.81	73.038
	F-measure	0.833			
LLCFS	Accuracy	76.27	0.631	78.31	74.70
	Sensitivity	71.08	1.388	75.37	68.23
	Specificity	81.40	1.005	82.69	76.80
	F-measure	0.800			
CFS	Accuracy	86.16	2.25	88.96	80.47
	Sensitivity	92.28	2.33	95.11	86.14
	Specificity	79.72	2.375	82.75	73.88
	F-measure	0.8517			
SVMRFE	Accuracy	71.92	0.832	74.43	69.93
	Sensitivity	66.90	1.493	71.14	63.53
	Specificity	76.68	1.187	79.61	72.88
	F-measure	0.7762			

**TABLE 8:** 10-fold cross-validation binary mean classification performance for MCI against AD using LSVM classifier using different feature selection methods.

**Feature selection method**	**Performance metrics**	**Mean (%)**	**Standard deviation**	**Max (%)**	**Min (%)**
LASSO	Accuracy	**97.80**	0.9862	98.32	91.99
	Sensitivity	**97.62**	1.0065	97.92	91.73
	Specificity	**97.74**	1.0720	98.5	92.07
	F-measure	0.98			
FSASL	Accuracy	83.71	0.90	85.00	78.15
	Sensitivity	90.63	1.57	92.23	84.12
	Specificity	77.10	1.098	79.74	72.55
	F-measure	0.838			
LLCFS	Accuracy	90.04	1.43	92.02	86.07
	Sensitivity	90.23	1.43	91.94	86.74
	Specificity	90.09	1.77	92.52	84.62
	F-measure	0.903			
CFS	Accuracy	84.41	1.95	86.81	79.18
	Sensitivity	90.37	2.14	93.26	83.73
	Specificity	78.10	1.90	80.79	72.84
	F-measure	0.83			
SVMRFE	Accuracy	82.87	0.903	83.96	78.832
	Sensitivity	81.68	1.22	84.34	77.10
	Specificity	84.11	0.94	85.21	80.72
	F-measure	0.854			

Tables 3–5 show the binary classification results using RELM classifier with five different feature selections. Results obtained through the feature selection methods are compared in regards to the performance metrics such as accuracy, sensitivity specificity and f-measure. Table 3 summarizes the AD versus HC classification. The LASSO feature selection method outperforms all other methods consider with the highest mean accuracy of 87.72%, mean specificity of 90.93% and mean sensitivity of 84.52%. Additionally, the standard deviation of LASSO is 0.4 which is less than less than 1. Similarly, the classification results of AD versus MCI and NC versus MCI using RELM are shown in Tables 4, 5. As shown in Table 4, the highest mean accuracy is 96.11 (±0.859) for HC against MCI classification and 93.86 (±0.766) for MCI against AD classification. The standard deviation is less than 1 in both mean classifications. Additionally the F-score is high in all three classifications (0.883 for HC against AD, 0.973 for HC against MCI, 0.968 for AD against MCI) using LASSO feature section method compared to other feature selection methods. The value of standard deviation less than one indicates that the data points of accuracy estimated tend to be close to the mean. Hence from the result it is very evident that the less inflated accuracy can be obtained using LASSO. Similarly, the high F-score indicates precision of classification is high compared to other feature selection methods.

Similarly, the comparison of classification of HC, MCI and AD using LSVM classifier with different feature selection methods are shown in Tables 6–8. Similar to RELM, the highest performance result in terms of mean accuracy, specificity, sensitivity and F-score was obtained by using LASSO for all three classification tests. As shown in Table 6, we obtained the accuracy of 90.63% specificity of 94.315% and sensitivity of 87.95% and F-score of 0.958 for AD against HC. In Table 7 the highest mean accuracy, specificity, sensitivity and F-score are obtained as 98.9, 99.68, 98.11, and 0.9856% for HC against MCI classification. Similarly, Table 8 shows the classification performance of AD against MCI. The highest mean accuracy, specificity, sensitivity and F-score are 97.81, 97.62, 97.74, and 0.98%.

From all these results, it is clearly evident that the use of LASSO as feature selection method is ideal choice for the classification using RELM and LSVM classifiers for the graph embedded data.

From Tables 3–5 the highest classification accuracies of RELM classifier using LASSO feature selection for AD against HC, HC against MCI and MCI against AD are 87.723% (±0.468), 96.11% (±0.859), and 93.86 %(±0.766). Similarly, from Tables 6–8 the highest classification accuracies of RELM classifier using LASSO feature selection for AD against HC, HC against MCI and MCI against AD are 90.63% (±0.515), 98.91% (±0.456), and 97.80% (±0.9862).

Now, the comparison of performance between two classifiers shows that the SVM can classify the given dataset more accurately with the highest mean accuracy for all three binary classifications. However, the small standard deviation of the classification HC against MCI and MCI against AD suggest that the classification accuracy values are less inflated in RELM as compared to LSVM.

**FIGURE 4:**
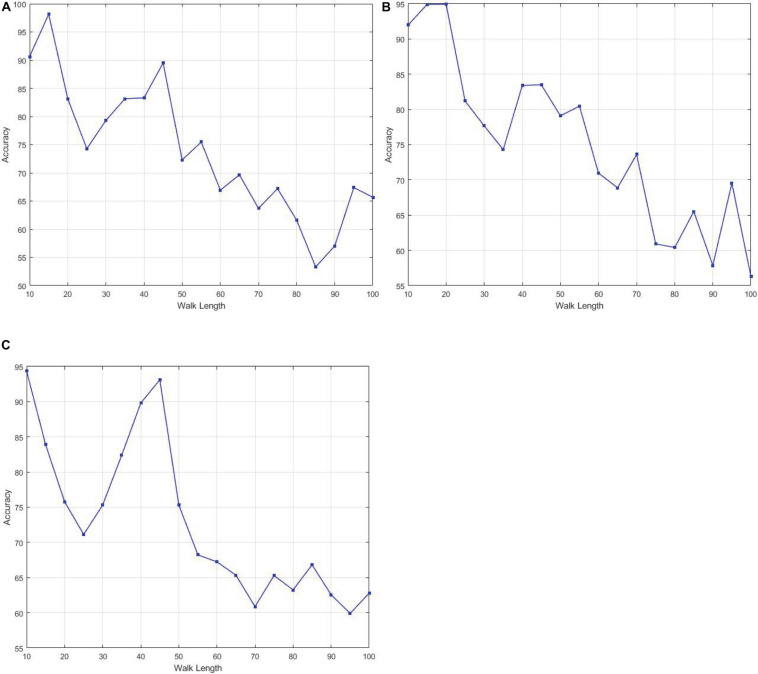
The effect of different parameter values of Walk Length of Node2vec on performance **(A)** AD against HC, **(B)** HC against MCI, **(C)** AD against MCI.

The number of hidden layer nodes influences the performance of the RELM classifier. In our experiment, we found 1000 number of hidden layer generated the best performance in terms of accuracy. Similarly, for SVM we set the default parameter defined for the MATLAB library. We performed the classification by varying different parameters on node2vec graph embedding. Figure 4 shows the effect of different parameters of node2vec on the performance of RELM classifier. We varied the walk length of node2vec from 10 to 100. In all experiments, increased value of walk length decreases the performance of classifier. For this experiment, we fixed two other parameters, dimension and number of walks to 32 and 200. Similarly, we set the parameters *p* and *q* to correspond localized random walks. With the smaller value of *p* and larger value of *q*, the random walk is easy to sample to the high-order -order proximity. Thus, we selected *p* and *q* randomly and performed graph embedding with *p* = 0.1 and *q* = 1.6.

## Discussion

Several studies based on rs-fMRI have been carried out for the classification of AD and MCI from HC subjects. Binary classification in combination of different classifier with different feature measure reported the accuracy ranging from 85 to 95% for AD against HC and 62.90 to 72.58% to and MCI against HC as shown in Tables 9, 10. These studies used the same MCI and HC subjects from the ADNI2 cohort. One can clearly notice that the number of subjects directly influences the accuracy. As the number of subjects increase the accuracy is decreased. As reported in previous section the highest accuracy for the classification of AD from is obtained in proposed work is 90.63% using the combination of LASSO and LSVM. If we compare the results for MCI against HC, the results obtained in current study outperform all the state of art methods. However, it is not fair to compare performance with other studies directly because each work employ different datasets, preprocessing pipelines, feature measures, and classifiers. Majority of works including (Eavani et al., 2013; Leonardi et al., 2013; Wu et al., 2013; Wee et al., 2014; Suk et al., 2016) have used subjects less than or nearly equal to 30 in each subject class. The main reason behind small number of dataset is the availability of fMRI data in ADNI2 cohort. All of these studies performed classification and made conclusion. Likewise, we also conducted our study using ADNI2 cohort with nearly equal number of subjects with previous studies and the cross validation was also done using these dataset.

**TABLE 9:** Comparison of performance of binary classification AD against HC with state of the art methods using rs-fMRI.

**Dataset**	**Feature measures**	**Classifier**	**Accuracy (%)**	**Reference**
AD:77, HC: 173	Combination of FC matrices, FC dynamics, ALFF	AUC	85	de Vos et al., 2018
AD: 12, HC: 12	Difference between DMN and SN map	LDA	92	Zhou et al., 2010
AD: 34, HC: 45	Graph measures	Naïve Bayes	93.3	Khazaee et al., 2017
AD: 15, HC: 16	Averaged voxel intensities of core regions in resting state networks: DMN, DAN, VAN	Multivariate ROC	95	Wu et al., 2013

**TABLE 10:** Comparison of performance of binary classification MCI against HC with state of the art methods using rs-fMRI.

**Dataset**	**Feature measures**	**Classifier**	**Accuracy (%)**	**References**
MCI: 31, HC: 31	Functional activity co-variations of ROIs	SVM	62.90	Eavani et al., 2013
MCI: 31, HC: 31	Group sparse representation	SVM	66.13	Wee et al., 2014
MCI: 31, HC: 31	SDFN	SVM	70.97	Leonardi et al., 2013
MCI: 31, HC: 31	Deep auto encoder and HMM	SVM	72.58	Suk et al., 2016

*FC, functional connectivity; ALFF, amplitude of low-frequency fluctuation; AUC, area under the curve; DMN, default mode network; SN, salience network; LDA, linear discriminant analysis; ROC, receiver operating characteristic; ROI, region of interest; AAL, automated anatomical labeling; DMN, default mode network; SDFN, sliding window-based dynamic functional network; HMM, hidden markov model.*

Mild cognitive impairment is a transitional stage between the healthy non dementia and dementia stage^2^. This stage is further divided into early MCI (EMCI) and late MCI (LMCI), according to extent of episodic memory impairment. The risk conversion from MCI to AD is higher in LMCI than in EMCI. In this study, we included only EMCI subjects in MCI group. The MCI converted and non-converted to is classified according to CDR and MMSE score. MCI subjects whose CDR undergoes change from 0.5 to 1 and MMSE score goes below 26 in subsequent visits are considered to have fulfilled the criteria to be MCI converted. In our study majority of subjects fulfill to be non-converted MCI. Only few subjects either have changed CDR score or MMSE score during the visits in the interval of 3, 6, 12, and 18 months. Additionally, none of the MCI subjects are recorded in the list of AD subjects.

### Limitations

While this study is focused on the stage diagnosis of AD progression using fMRI alone using ADNI2 cohort, the major limitation of this study is the limited sample size of ADNI2 (31 AD, 31 MCI, and 31 HC). In this context, the entire population is not represented adequately with the dataset we used. Thus, we cannot guarantee the generalization of our results to other groups.

## Conclusion

It is widely accepted that the early diagnosis of AD and MCI plays an import role to take preventive action and to delay the future progression of AD. Thus the accurate classification task of different stages of AD progression is essential. In this study, we demonstrated graph based features from functional magnetic resonance (fMR) images can be used for the classification of AD and MCI from HC. Additionally, we used multiple feature selection techniques to cope with the smaller number of subjects with larger number of feature representations. The appropriate amount of features is extracted from standard Alzheimer’s disease Neuroimaging Initiative cohort that lead to maximal classification accuracies as compared to all other recent researches. Among different feature section methods LASSO together with LSVM on graph based features significantly improved the classification accuracy.

## Data Availability Statement

The raw data supporting the conclusions of this article will be made available by the authors, without undue reservation.

## Ethics Statement

The studies involving human participants were reviewed and approved by Alzheimer’s Disease Neuroimaging Initiative (ADNI). The patients/participants provided their written informed consent to participate in this study.

## Author Contributions

RL has generated the idea and conducted the experiments. G-RK has reviewed idea and final verification of results. All authors contributed to the article and approved the submitted version.

## Conflict of Interest

The authors declare that the research was conducted in the absence of any commercial or financial relationships that could be construed as a potential conflict of interest.

## Footnotes

1 http://adni.loni.usc.edu/

2 https://www.nia.nih.gov/alzheimers/clinical-trials/alzheimers-disease-neuroimaging-initiative-2-adni2

3 http://www.restfmri.net

4 http://www.fil.ion.ucl.ac.uk/spm
